# Risks and opportunities for children's well-being in resource-constrained multigenerational households during COVID-19: Implications for school psychology interventions

**DOI:** 10.1177/01430343221144407

**Published:** 2022-12-18

**Authors:** Kamleshie Mohangi

**Affiliations:** Department of Psychology of Education, 274781 University of South Africa, Pretoria, South Africa

**Keywords:** child well-being, COVID-19, multigenerational family, resource-constraints, risk and opportunity, school psychology

## Abstract

The coronavirus disease (COVID-19) pandemic had a global impact on family social and economic well-being. Individuals and families sought alternative living arrangements as a result of the financial crisis, health implications, and housing insecurity, with many joining multigenerational households. However, it is unknown how multigenerational family life affects children's well-being. Therefore, this qualitative study explored risks and resilience-building opportunities for children's psychological and social well-being in resource-constrained multigenerational households during the COVID-19 pandemic in South Africa. Five multigenerational families were selected through snowball sampling and case design. The three generations of participants were grandparents (*n* = 5), parents (*n* = 7), and children (*n* = 4). Data were gathered through a questionnaire and interviews. The study received institutional ethics approval. After thematic analysis, two themes and six sub-themes were identified. Risks were related to interpersonal conflict, family collective fear of COVID-19, and children's multiple other fears. Opportunities were identified as academic support, shared responsibilities, life skills and values acquisition, and family cohesion. Results demonstrated the potential risks and resilience-building opportunities multigenerational households present for children's psychosocial well-being. Multisystemic influences in a multigenerational household contribute to children's adjustment. These outcomes necessitate systemic school psychology interventions. Longitudinal studies are recommended to explore child well-being trends in multigenerational households in varying socioeconomic contexts.

## Introduction

Research has established that the coronavirus disease (COVID-19) pandemic (hereafter referred to as ‘the pandemic’) impacted the socioeconomic welfare of families worldwide (Alonzo et al., 2021; Batra et al., 2022; Gittings et al., 2021; Spinelli et al., 2021; Thibodeau-Nielsen et al., 2021). To contain the spread of COVID-19, countries applied lockdown, quarantine, isolation, and social distancing measures (Brooks et al., 2020; Naidu, 2020). However, the severe lockdown enforced in South Africa had significant economic repercussions, with business closures and loss of employment (Naidu, 2020; Posel et al., 2021). Cumulative financial stress and limited social support prompted mental health, food, and housing insecurity (Posel & Casale, 2020, 2021).

Large segments of the population in post-apartheid South Africa continue to face inequalities and live below the poverty line (Fouché et al., 2020; Naidu, 2020). Consequently, pre-COVID-19, crowded households with multigenerational families sharing resources were common in South Africa (Naidu, 2020). The pandemic-related lockdown and its consequences, on the other hand, exacerbated pre-existing socioeconomic vulnerabilities (Gittings et al., 2021; Naidu, 2020), prompting an increase in the number of people moving into multigenerational households to reduce living costs. Although this living arrangement had mutual benefits, it worsened the financial situation of already-constrained families (Posel & Casale, 2020, 2021).

While research has established that family support contributes to children's social, emotional, and cognitive growth (Department of Social Development (DSD), 2021), it is not yet known how multigenerational family life influences child well-being outcomes in South Africa. Therefore, this study explored possible risks and resilience-building opportunities affecting children's psychosocial well-being in multigenerational households during the pandemic. The research question was: What factors influence risks and resilience-building opportunities for children's psychosocial well-being in multigenerational households?

### The South African context and multigenerational households

The prolonged pandemic caused significant disruption to families’ lives. In March 2020, the World Health Organisation (WHO) declared COVID-19 a worldwide pandemic, and South African authorities implemented lockdown measures (Fouché et al., 2020). Because of unemployment and financial difficulties, parents experienced profound stress (Alonzo et al., 2021; Posel et al., 2021; Prime et al., 2020), which increased when schools and childcare centres closed, necessitating home-based care and education (Evans et al., 2020). Young and single parents (Gittings et al., 2021; Iztayeva & Sciences, 2021) who lack domestic help were more likely to seek extended family support (Russell et al., 2020). Before the pandemic, communal households helped single parents, the elderly, and jobless relatives (Posel & Casale, 2021) and, therefore, were common in many nations, including South Africa (StatsSA, 2020), Australia (Easthope et al., 2017), USA (He & Jia, 2021), UK (Cantillon et al., 2021), India (Buheji & Buheji, 2020), and China (Zeng & Xie, 2014).

According to Statistics South Africa (StatsSA) (2020, p. 6), households are “individuals who live together under the same roof or in the same yard and share resources such as food or money”. A multigenerational household typically includes three generations of related adults and children living together (Burgess & Muir, 2020; StatsSA, 2020), which, pre-COVID-19, comprised approximately 14.9% of South African households (StatsSA, 2020).

According to Posel and Casale (2021), financial limitations increased families’ transition into multigeneration homes during the pandemic. Reports indicate that during the first few months of the lockdown in 2020, almost 16% of adults in South Africa moved into different households, motivated by the need for economic, social, and housing security (Gittings et al., 2021; Posel & Casale, 2021).

In South Africa, family structures like single-parent, child-headed, and multigenerational households substantially influence family functioning (DSD, 2021). For example, a family that provides individuals with social identity, economic support, nurturing, socialisation, and security promotes effective adaptation to a larger society (October et al., 2022). In other words, families that work together successfully (to build resilience) promote healthy family outcomes (resilience) (Walsh, 2016). Thus, this paper positions resilience building as a process (Masten, 2018).

### Risks and opportunities for children

Multiple factors within multigenerational households pose risks to children. It is established that family financial difficulties were a significant source of household stress during the pandemic. Additionally, sharing a home without privacy aggravated interpersonal interactions (Prime et al., 2020). Adults who experience elevated stress levels resort to poor coping mechanisms (Spinelli et al., 2021). Moreover, overcrowding, changes in family structure, household expectations and daily family routines are disruptive and confusing for children (Bates et al., 2021; Prime et al., 2020; Shorey & Ng, 2020). Environmental stress contributes to children's behavioural problems (Samji et al., 2022), increases gender-based and family violence (Govender et al., 2020; Usher et al., 2021) and places children at risk for abuse (Fouché et al., 2020; Usher et al., 2021). Of concern is that when children witness domestic violence, they are emotionally and psychologically harmed (Cluver et al., 2020; Rider et al., 2021).

Despite challenges, studies report the benefits of multigenerational living (Easthope et al., 2017; Evans et al., 2020; He & Jia, 2021). According to Walsh (2020), when households include extended family members, it demonstrates how individual and family resilience pathways may be forged. For example, Burgess and Muir (2020) observed that children in multigenerational homes acquired resilience-building social skills. Others found that cohabitation facilitated eldercare and childcare (He & Jia, 2021) and relieved children's loneliness (Loades et al., 2020; Shorey & Ng, 2020). Therefore, family support is crucial in children's emotional and cognitive growth (DSD, 2021; StatsSA, 2020, p. 11). Grandparents offer childcare (Dunifon et al., 2018), contributing to children's academic growth and social competence (Edwards, 2015; Wild, 2018) and mitigating children's behavioural difficulties. At the same time, their instrumental support is evidenced in household duties and caring, while their expressive support is demonstrated in comfort and nurturance (Gilligan et al., 2020).

## Theoretical understanding

The pandemic posed complex and multisystemic threats at the child, family, school, and community levels. Although this paper focuses on children in family households, broader systemic impacts are equally significant since families functioning in supportive multisystemic environments are critical in improving individual and societal outcomes (DSD, 2021). Adopting Bronfenbrenner's (1979, 2005) well-established (bio)ecological systems approach and adaptations (see Bronfenbrenner & Morris, 1998) gives insight and understanding into how child and family multifaceted relationships during the pandemic influence resilience outcomes. Such models suggest that the interdependency of interacting systems and sub-systems have reciprocal effects that influence change, growth, and development.

The process of fostering resilience is critical, not only for children but in relation to the proximal systems in which they are involved. Following Masten and Motti-Stefanidi (2020, p. 98), ‘resilience is best defined as a systems concept referring to the successful adaptation of a complex dynamic system to threats or disturbances, drawing on distributed capacity through many processes.’ The premise is that intrapersonal, interpersonal, organisational, community and policy factors impact well-being at the micro-, meso-, exo-, macro- and chronosystem levels (Bronfenbrenner, 1979). Person qualities, proximal spaces, and context (family microsystem level) (Bronfenbrenner & Morris, 1998) influence psychological functioning (emotions and behaviour) and social experiences (relations, tradition, and culture). In this way, school, community, and state intersection processes also influence family decisions and play a role in children's adjustment (Pocock et al., 2012). During the pandemic, state macrosystems at the organisational level regulated restrictions and impacted socioeconomically disadvantaged individuals, families, and communities (Cluver et al., 2020).

### School psychology in South Africa

Schools are vital links to children's social networks, and with the pandemic-related school closures, loss of social contact adversely affected children's psychosocial adjustment (Loades et al., 2020; Samji et al., 2022; Semo & Frissa, 2020). Consequently, school psychologists must lead systemic support to children, families, and the community post-pandemic (Swart & Eloff, 2018). In South Africa, school psychologists are referred to as educational psychologists (Jimerson et al., 2009), a registration category mandated by the Health Professions Council of South Africa (HPCSA). Historically, marginalised communities in South Africa did not have access to psychological services (Swart & Eloff, 2018). Following an inclusive education policy implementation (Department of Education (DoE), 2001), public schools receive educational psychology services via district-based support teams. However, considering the large number of children requiring psychosocial care during and after the pandemic, there is an acute need for more educational psychologists to support under-resourced communities.

## Method

### Sampling and participants

Snowball sampling was used to sample multigenerational households (family units) (Parker et al., 2021; Woodley & Lockard, 2016). This strategy facilitates access to ‘hard-to-reach’ populations (Woodley & Lockard, 2016). In this study, the researcher's academic colleague identified one family she knew that fulfilled the study's inclusion criteria. The researcher telephonically approached the mother of that family and explained the research project. This family then identified another family unit that met similar inclusion criteria. In this manner, the sampling technique identified five family units and individual participants who met the inclusion criteria:

1. Family units comprised three generations (grandparent, parent, child).
2. Individuals lived in multigenerational households during the pandemic.

All five grandmothers, mothers and two fathers gave personal consent and voluntarily participated. Four parents consented to their minor children's participation in the study. For contextual background, demographic information is presented in Table 1.

**Table 1.** Demographic information and participant code.

	Household composition	Source of income	Participant and code
**FAMILY A**	Maternal grandmother (69 years) Mother (34 years) Child A (9 years) Relative (unemployed) Relative's child (10 years) Relative's baby (3 months)	Grandmother's old-age pension Mother – part-time employment	Maternal grandmother A Mother A Child A (female – 9 years)
**FAMILY B**	Maternal grandmother (72 years) Mother (36 years) Child 1 (10 years) Child 2 (3 years)	Grandmother's old-age pension Mother – part-time employment	Maternal grandmother B Mother B Child B (female-10 years)
**FAMILY C**	Maternal grandmother (65 years) Mother (39 years) Father (44 years) Child 1 (11 years) Relative (unemployed)	Mother and father are employed.	Maternal grandmother C Mother C Father C Child C (male-11 years)
**FAMILY D**	Paternal grandmother (66 years) Paternal grandfather (73 years) Mother (40 years) Father (48 years-unemployed) Child 1 (15y ears) Child 2 (12 years) Relative (unemployed)	Grandparents’ old-age pensions Mother – part-time employment	Paternal grandmother D Mother D Father D Child D (male-15 years)
**FAMILY E**	Paternal grandmother (69 years) Paternal grandfather (75 years) Mother (42 years-unemployed) Father (50 years-unemployed) Child 1 (10 years) Child 2 (8 years) Baby (4 months)	Grandparents’ old-age pension Father – casual work	Paternal grandmother E Mother E

*Source*: Primary data.

### Research sites

Participating families were sampled from two low-income, semi-urban neighbourhoods in Gauteng, South Africa. Low-income areas are characterised by poor infrastructure, lack of service delivery and limited access to essential healthcare facilities, and good-quality schools (Pernegger & Godehart, 2007). Unemployment and poverty in underprivileged communities can lead to the formation of multigenerational households (Posel & Casale, 2021).

### Materials

*a) Demographic questionnaire:* The research assistant, an educational psychologist, administered the questionnaires telephonically. One adult participant in each family voluntarily completed the questionnaire. Questions pertained to the family unit's composition, age range, gender, relationships, children's grades at school and adult employment status.

*b) Semi-structured interviews:* Telephonic, online, and face-to-face interviews were conducted between July and December 2021.

*Adult interviews:* Questions explored participants’ perspectives on multigenerational family living, potential risks and benefits to children and general views on the pandemic's implications for their families and themselves. All 12 adults preferred telephonic interviews. Each interview lasted 20–30 minutes and was scheduled for participant suitability.

*Child interviews:* Child interviews were conducted in an informal conversational manner to minimise anxiety related to participation (Cohen et al., 2000). Four children aged 9 to 15 participated in individual interviews in their parents’ presence as minors. Three interviews were conducted via the Zoom platform (parent's cell phone) and lasted between 10 and 20 minutes. The 15-year-old adolescent participant preferred a face-to-face interview. The participant and parent chose a quiet space on a playground next to their house and within the parent's view for the interview.

### Ethical measures

Various measures were taken to ensure the research was conducted ethically. Written permission to conduct the study was obtained from the institution's Research Ethics Committee (2020/07/08/90188527/21/AM). Standard ethics principles of voluntary participation, protection from harm, confidentiality, and privacy were adhered to throughout the study. Although precautionary measures were taken, the potential risks from online communication were discussed with participants. Participants provided informed consent (adults) and assent (children) after the aim and details of the study were explained. Measures were taken to ensure the participants understood the research and were comfortable participating. Parents assisted their children with the process of informed assent. All participants were invited to discuss any uncertainties with the researcher or research assistant. Moreover, all participants consented to have their interviews digitally recorded. Online and face-to-face interviews were documented in field notes. Member-checking was conducted telephonically. A coding process safeguarded the identity and confidentiality of individuals and family units.

### Design

This qualitative study employed an exploratory multiple-case design with an interpretivist paradigm. This approach suited the nature of the study as it offered a comprehensive account of a phenomenon (multigenerational household) and facilitated an understanding of individuals’ interpretation of their life experiences (Creswell & Poth, 2018).

### Data analysis

Data were analysed according to Braun and Clark’s (2006) six phases of thematic analysis. Phase 1 required the researcher to become familiar with the data sets (interviews and questionnaires). Participants’ responses were transcribed verbatim for accuracy. In Phase 2, the transcriptions, field notes and questionnaire were analysed. Phase 3 entailed searching for codes across the data sets, grouping codes into clusters and identifying possible themes and sub-themes. In Phases 4 and 5, themes were revised and consolidated into two main themes and six sub-themes. In Phase 6, themes and sub-themes were organised to support the research question. During the analytic process, the researcher was cognizant of Braun and Clark's (2019) assertion that themes are ‘interpretive stories about the data’ and was, therefore, ‘reflective and thoughtful’ throughout the process (Braun & Clark, 2019, p. 7).

Furthermore, to obtain a more nuanced reading of the data, another qualitative researcher collaborated with the analytic process by reading and re-reading the transcripts (Braun & Clark, 2019). Ultimately, detailed descriptions aimed to contribute to the rigour of the study.

## Results

Two themes and six sub-themes were developed from the analysis.

### Theme one: factors that posed risks to children's well-being

***Interpersonal conflict:*** Living in close confines with relatives across generations in a crowded household contributed to interpersonal conflict. Adult quarrels about distributed household tasks were identified as sources of unhappiness for children. Additionally, children's exposure to family disagreements contributed to their feelings of anxiety. For example, Child A claimed:

They fight every day, my mother and grandmother [sic]. My mother said my grandmother she was lazy and not helping [sic]. This makes me worry.

Parent discord arising from unemployment, finances, alcohol abuse, and hostility, had a detrimental impact on children's well-being since they were reprimanded aggressively. According to Mother E:

My husband does not work now but wants all his meals and money from me and his mother [sic]. It is very bad when he drinks [alcohol]; he gets angry and shouts. Sometimes he smacks the child and the baby cries when there is shouting.

Sharing limited space created friction. In some cases, family members resented sharing their homes with extended family members. Mother A explained:

We share the bedroom because my sister is here with her children. We buy food for the baby, but he cries a lot. The house is very noisy, and there is lots of shouting. My child gets upset.

A significant factor in parent–child conflict is limited finances. In this case, it is claimed that children have little understanding of the family's financial circumstances. Mother A outlined her difficulties:

I am the only one here getting any money – but my mother gives us some of her grant. This child wants everything … She doesn’t understand that there is not enough money to pay the rent and electricity. I get very angry with everyone, my mother, my daughter and even my sister [sic].

Family conflict is exacerbated when household responsibilities are inequitably distributed. For instance, when one family member is perceived to be burdened without assistance, it leads to resentment, as was stated by Child D:

It's not fair at home. My mother gets tired, and I help her with everything, but my aunt does nothing at home.

When responsibilities are shared, the environment is calm, and children are relaxed. This viewpoint is shared by Mother D whose perspective differed from her son's (Child D) when she showed acceptance of the family's circumstances.

My mother-in-law is helpful and kind to me when I have to cook for the family. She supports me. But it's the boys who help me the most. My husband's sister doesn’t help. But I don’t mind. The children are happy with things here.

***Family fear of COVID-19:*** Family members feared contracting COVID-19 and its implications for the family. A recurring concern related to family finances and children's care. Grandparents were concerned about hospital costs and food provision for their grandchildren in the event of their hospitalisation. Grandmother B expressed her distress:

I am very scared. If I catch the disease, who is going to care for me and pay the hospital [costs][sic]. I get a grant [old-age pension], and I sell some vegetables, but there will be no income if I go to the hospital. My children and grandchildren will starve.

Parents were concerned about transmitting COVID-19 to grandparents since grandparents contributed to the family's finances. Mothers, on the other side, were concerned that if a grandmother became ill, they would not be able to care for their children. This complex dilemma demonstrated that the mothers’ worry was their parents’ utility for their children's care. Mother B stated:

I worry my mother will get sick. She is 72 [years old] and has high blood pressure. She helps me with my children and cooks for them. It will be very hard [difficult] for me if she gets sick. I will have to look after the children myself, and they make me angry.

***Children's fears:*** Children expressed multiple fears*.* For example, Child C was concerned about the virus, the deployment of the army, and the fear of grandparents dying. In-residence grandparents play a significant part in children's lives, developing a solid bond and causing children to fear losing them. Child C explained:

This COVID-19 is very scary. But I was more scared when I saw the army here. They were pushing people[sic]. I am very worried my gogo [grandmother] will die. Who will take care of us then? [sic]

Another common concern was children's schooling. Child D expressed: *I am worried about going back to school. I don’t think I will pass the year. Also, my friends won’t know me anymore. I wish I could stay at home.* At the time of this study, schools were partially opened, and children were conflicted about schools reopening fully. As a result of parents’ perceptions that their children were bored, irritated, and getting into trouble, the tension between generations exacerbated their disposition. Mother B explained:

These children are causing too much problems here at home[sic]. They won’t listen about social distancing. Then they are angry.

### Theme two: opportunities for children's well-being

***Children received academic and technology assistance:*** Children benefitted from in-house relatives’ help when their parents were unavailable. This form of support elevated children's academic progress during school closures. Child A:

Granny reads to me every night. I love her to read, and she has a nice voice-very soft [sic]. My cousin in grade 4 also helps me to read.

When parents were not available to help children, extended family relatives provided technological support such as access to the computer and internet. For example, Child C explained:

I have to do homeschooling. The problem is I need help, and my mother and father work. My uncle is here and has no job. He has a laptop. So, he helped me to find information on Google [sic].

***Shared household responsibilities and acquired vital life skills*:** Members must contribute to shared household responsibilities for structure and order in the household. In the process, children learn valuable life skills that involve meal preparation, domestic work, childcare, and eldercare. Mothers and grandmothers shared this viewpoint.

Mother D: They help me with the housework and see their grandparents. I know that when they grow up, they will be able to care for me and themselves.

Grandmother A: We must teach the children, and it's our responsibility to see that they grow up well. I care a lot for them.

***Family cohesion promotes family values:*** A healthy multigenerational environment generates opportunities for children to emulate compassion, care, and empathy. Participants viewed providing care for their families as a sense of responsibility and commitment. For example, Grandmother A reflected on her role in maintaining good family relations to generate happiness:

I look after the children. I cook and wash all the clothes. It's hard, but I have no choice because I care about them and want our family to be happy.

Child D also said it was one's duty to care for family members*:*

It's my duty to help at home. I like to take care of my grandparents. COVID [COVID-19] is very bad, and I don’t want them to get sick. I try to do everything, so they all don’t go out [sic].

When grandparents express comfort and protection, children recognise it as care which they are keen to emulate. Child A acknowledges her grandmother's care and protection:

But I think my grandmother takes good care of me. She makes sure that I eat my meals and that I have snacks, and she does not shout or smack and hit me like my mother [does]. She shouts[reprimands] at my mother. She makes me pray with her, and I think God will help us.

## Discussion

This study aimed to identify potential risks and resilience-building opportunities that influence children's psychosocial well-being in multigenerational households during the pandemic. Insights from Bronfenbrenner's (1979, 2005) (bio)ecological systems perspectives, Bronfenbrenner and Morris’s (1998) emphasis on person, place, context, and time, resilience in adversity (Masten & Motti-Stefanidi, 2020) and family resilience (Walsh, 2016) guided understanding.

At the family microsystem level, research has established that pandemic-related adult stresses, mainly from financial concerns, were precursors to family interpersonal conflict (Prime et al., 2020; Spinelli et al., 2021). In this study, interpersonal conflict at the family microsystem level posed risks to children's emotional adjustment. For example, children were anxious when they witnessed parental aggression or violence. Studies show that when parents’ stresses are heightened during the pandemic, their mental and emotional coping resources are depleted (Brown et al., 2020; Prime et al., 2020), leading to conflictual relationships. Other studies confirm that verbal conflict and harsh discipline intimidate children (Brown et al., 2020; Lee et al., 2021), and the risk for abuse and neglect is high (Fouché et al., 2020). It is deduced from the study that adult mental health challenges, exacerbated by job losses or reduced income (Spinelli et al., 2021), precipitate inappropriate parenting (harshness or physical aggression) and related behaviours (alcohol misuse) with cascading effects on children's psychological health. For instance, when social isolation increases adult psychological distress and precipitates anger (Brooks et al., 2020). Moreover, as in this study, space proximity with confinement to an overcrowded house may have triggered adult stress responses (Mashaphu et al., 2021; Russell et al., 2020). Children are at risk of psychological maladjustment when parents cannot attend to children's physical, emotional and schooling needs (Maree, 2021; Thibodeau-Nielsen et al., 2021).

Systemically, physical health, stress, and anxiety are intrapersonal outcomes precipitated by larger state-regulated exo-, macro-, and chronosystem legislations. For example, South Africa's ‘hard lockdown measures’ precipitated dire socioeconomic consequences. Like in other countries, this affected interpersonal connections at meso- and microsystemic levels (Shorey & Ng, 2020).

The grandparent–child sub-system is an enabling family resource (Wild, 2018) and positive interpersonal family relations offer children protection and overall mental health adjustment (Uccella et al., 2021; Walsh, 2016, 2020). For example, during the pandemic, grandparents and extended family members supported children with online homework and caregiving (Evans et al., 2020; October et al., 2022). Furthermore, children are protected when cared for by co-resident grandparents in the microsystem (Wild, 2018). Positive caregiving and solution-focused coping strategies create resilience-building opportunities (Panda et al., 2020) and environments where children feel motivated to engage. For instance, co-residing relatives mediate adverse parenting and protect children. In many cases, grandmothers feel enabled and worthwhile when they share old-age pension money with jobless adult children, extending family finances (Cantillon et al., 2021). These results align with the socioecological model, which presupposes the dynamic interplay among environmental, personal, and relational factors (Stokols, 1992).

The child-peer relationship is another sub-system that influences resilience building. However, with school closure, decreased companionship aggravated children's loneliness (Loades et al., 2020; Samji et al., 2022) and placed them at risk for depression (Panda et al., 2020; Samji et al., 2022). When children lack physical activity, sleep irregularly, and spend more time with electronic devices, their anxiety increases (Uccella et al., 2021). Like other studies, children's fear and uncertainty about schools’ re-opening and passing grades were exacerbated when they worried about contracting and transferring the virus to grandparents (Rider et al., 2021). Of concern is that the long-term implications of chronic anxiety on children are not yet apparent. Rider et al. (2021) report that childhood trauma, such as the consequences of the pandemic, may not be evident until later in life, placing them at risk for mental health challenges. The chronosystem encapsulates the dimension of time and how it relates specifically to the interactions between these systems and their influences on child adjustment during the pandemic and after that.

Supporting resilience-building opportunities for child well-being in complex family environments requires understanding how events in one system might have immediate or long-term consequences for others (Stokols, 1992).

From the family systems perspective, resilient families (in this case, multigenerational) adapt according to family stresses (Walsh, 2016). Specific processes involved in improving families’ response to stress and fostering resilience included shared belief systems, organisation processes in the family, and communication. Furthermore, the family's adaptive systems’ protective function arises from routines and rituals (Harrist et al., 2019). Family belief systems ingrained in family values, culture, spirituality, and shared experiences are one way to enhance resilience (Masten & Motti-Stefanidi, 2020; Walsh, 2020; Ting et al., 2021). Likewise, Ting et al. (2021) found that cultural responses and religious beliefs moderate everyday stress and major life crises such as the effects of the pandemic. Similarly, religious and cultural events in South Africa lie at the heart of communities’ social systems and contribute to a sense of connectedness and belonging (Mashaphu et al., 2021).

Family cohesion and connectedness, associated with mutual support, commitment, and collaboration, are critical organisational processes in family resilience (Walsh, 2016). These features were evident in one family during the current study. Notably, family members must express their emotions openly since mutual empathy strengthens family connections (Walsh, 2020). Although the South African White Paper on Families (DSD, 2021) underscores the family's role in nurturing, supporting, and socialising its members, this perspective demands agency and deliberate action in all household compositions and structures.

During the COVID-19 pandemic, the notion of space and time was critical. For example, how can the protection offered by multigenerational homes be sustained over time? Knowing that the effectiveness of proximal spaces is minimised in unstable and unpredictable contexts across space and time (Swart & Pettipher, 2016), are the specific risks identified in this study transient and applicable only to the pandemic? The aim should be the sustainability of family-generated opportunities for child well-being beyond the COVID-19 pandemic.

### Limitations

Limitations are noted in this study. First, the research sites were restricted to two communities in resource-scarce environments in Gauteng. Sampled families represented those who felt comfortable with participation. Participants were primarily female. Therefore, the extent to which findings can be generalised to a larger sample of families from diverse cultures, ethnicities, educational backgrounds, experiences, and income levels may be limited. However, detailed descriptions may facilitate replicability. Interviews conducted via technology such as cell phones and Zoom could have restricted sharing of personal information. Lastly, children's responses could have been limited due to the parent's presence.

### Implications

Despite the limitations, findings from this study have meaningful implications for children worldwide. The results confirm that although multigenerational households offer resilience-building opportunities for children's psychosocial well-being, inherent risks require intervention.

Within the family microsystem, the quality of bi-directional relationships bounded by context, time and support may determine child resilience outcomes. Despite contextual and cultural differences, children in other countries may face similar opportunities and threats (Samji et al., 2022; Spinelli et al., 2021; Uccella et al., 2021), and therefore, the findings from this study could have international relevance.

Accordingly, school psychologists are ideally placed to fulfil their roles in supporting children, families, and the community through effective multisystemic intervention. In this manner, they execute the United Nations 17 Sustainable Development Goals (SDG) to create a better society for all (The Lancet Public Health, 2020). School psychologists may make a significant contribution to the implementation of SDG 3 (good health and well-being for everyone), SDG 4 (inclusive and equitable quality education), and SDG 10 (reduced inequalities). These goals are inextricably interconnected in multiple social systems for resilience-building.

### Recommendations for school psychology interventions

Based on the study's findings, recommendations for global application are made.

*Build on opportunities to mitigate risk.* School psychologists may capitalise on multigenerational family life benefits to assist children's adjustment. For instance, focused efforts on improving family values and communication skills could alleviate communication-related conflict.

*Knowledge and awareness of diverse cultures.* School psychologists must be sensitive to families’ cultural backgrounds because each family's experience would differ (Edwards, 2015; Evans et al., 2020). The school psychologist must have intervention options when addressing the family's unique systemic challenges. For example, when the school psychologist and the child's primary language differ, non-verbal play-based therapeutic measures may be used (Thibodeau-Nielsen et al., 2021).

*Social support.* School psychologists could help children re-enter social groups or establish new friendships to extend their support networks to alleviate school-related apprehension.

*Learning support:* Grandparents and in-house relatives help to provide learning support to children under the class teacher's supervision.

*Psychoeducation*. School psychologists can connect family members with adult mental health support organisations for stress management and coping skills interventions. One such group is SADAG (South African Depression and Anxiety Group).

## Conclusion

This study contributes to understanding risk factors and resilience-building opportunities for children's well-being in multigenerational households during the pandemic. Interpersonal family conflict, the family's collective fear of COVID-19 and children's multiple other fears were identified as risk factors contributing to maladjustment and restricting well-being. This finding is supported by previous research (Gilligan et al., 2020). In contrast, the study found that when children received educational assistance, shared household tasks and experienced family cohesion and nurturing, they acquired vital life skills and family values that offer opportunities for well-being and resilience-building.

Systemically implemented school psychology interventions may reduce adversity risks and promote children's psychosocial development. These recommendations are applicable worldwide. While this study was undertaken during the pandemic, the findings are likely relevant to other global adversities. Future longitudinal studies might explore trends in factors that present risks and opportunities in multigenerational households constituting varying ages, genders, and socioeconomic contexts.

## References

[bibr1-01430343221144407] Alonzo D. Popescu M. Ioannides P. Z. (2021). Mental health impact of the COVID-19 pandemic on parents in high-risk, low-income communities. International Journal of Social Psychiatry, 68, 575–581. 10.1177/0020764021991896 33517821 PMC7862916

[bibr2-01430343221144407] Bates C. R. Nicholson L. M. Rea E. M. Hagy H. A. Bohnert A. M. (2021). Life interrupted: Family routines buffer stress during the COVID-19 pandemic. Journal of Child and Family Studies, 30(11), 2641–2651. 10.1007/s10826-021-02063-6 34404970 PMC8360776

[bibr3-01430343221144407] Batra K. Pharr J. R. Terry E. Labus B. (2022). Assessing psychological impact of COVID-19 among parents of children returning to K-12 schools: A U.S. based cross-sectional survey. Healthcare, 10, 775. 10.3390/healthcare10050775 35627912 PMC9141861

[bibr4-01430343221144407] Braun V. Clarke V. (2006). Using thematic analysis in psychology. Qualitative Research in Psychology, 3(2), 77–101. 10.1191/1478088706qp063o

[bibr5-01430343221144407] Braun V. Clark V. (2019). Reflecting on reflexive thematic analysis. Qualitative Research in Sport, Exercise and Health, 11(4), 589–597. 10.1080/2159676X.2019.1628806

[bibr6-01430343221144407] Bronfenbrenner U. (1979). The ecology of human development. Harvard University Press.

[bibr7-01430343221144407] Bronfenbrenner U. (2005). Making human beings human: Bioecological perspectives on human development. Sage.

[bibr8-01430343221144407] Bronfenbrenner U. Morris P. A. (1998). The ecology of developmental processes. In Lerner R. M. (Ed.), Handbook of child psychology (5th ed., Vol. 1, pp. 993–1028). Wiley.

[bibr9-01430343221144407] Brooks S. K. Webster R. K. Smith L. E. Woodland L. Wessely S. Greenberg N. Rubin G. J. (2020). The psychological impact of quarantine and how to reduce it: Rapid review of the evidence. The Lancet, 395(14), 912–920. 10.1016/S0140-6736(20)30460-8 PMC7158942 32112714

[bibr10-01430343221144407] Brown S. M. Doom J. R. Lechuga-Peña S. Watamura S. E. Koppels T. (2020). Stress and parenting during the global COVID-19 pandemic. Child Abuse & Neglect, 110, 104699. 10.1016/j.chiabu.2020.104699 32859394 PMC7440155

[bibr11-01430343221144407] Buheji M. Buheji A. (2020). Intelligent living with ‘ageing parents’ during COVID-19 Pandemic. International Journal of Psychology and Behavioral Sciences, 10(3), 76–83. 10.5923/j.ijpbs.20201003.03

[bibr12-01430343221144407] Burgess G. Muir K. (2020). The increase in multigenerational households in the UK: The motivations for and experiences of multigenerational living. Housing, Theory and Society, 37(3), 322–338. 10.1080/14036096.2019.1653360

[bibr13-01430343221144407] Cantillon S. Moore E. Teasdale N. (2021). COVID-19 and the pivotal role of grandparents: Childcare and income support in the UK and South Africa. Feminist Economics, 27(1–2), 188–202. 10.1080/13545701.2020.1860246

[bibr14-01430343221144407] Cluver L. Shenderovich Y. Meinck F. Berezin M. N. Doubt J. Ward C. L. Parra-Cardona J. Lombard C. Lachman J. M. Wittesaele C. Wessels I. Gardner F. Steinert J. I. (2020). Parenting, mental health and economic pathways to violence prevention against children in South Africa. Social Science and Medicine, 262, 113194. 10.1016/J.SOCSCIMED.2020.113194 32763649 PMC10782842

[bibr15-01430343221144407] Cohen L. Manion L. Furbush D. (2000). Research methods in education (5th ed). Routledge Farmer.

[bibr16-01430343221144407] Creswell J. Poth C. (2018). Qualitative inquiry & research design: choosing among five approaches. Sage Publications.

[bibr17-01430343221144407] Department of Education (DoE). (2001). *Education White Paper 6. Special needs education: building an inclusive education and training system*. Department of Education.

[bibr18-01430343221144407] Department of Social Development (DSD) Republic of South Africa. (2021, March 31). *Revised white paper on families in South Africa* (No. 44799). www.gpwonline.co.za.

[bibr19-01430343221144407] Dunifon R. E. Near C. E. Ziol-Guest K. M. (2018). Backup parents, playmates, friends: Grandparents’ time with grandchildren. Journal of Marriage and Family, 80(3), 752–767. 10.1111/jomf.12472

[bibr20-01430343221144407] Easthope H. Liu E. Burnley I. Judd B. (2017). Changing perceptions of the family: A study of multigenerational households in Australia. Journal of Sociology, 53(1), 182–200. 10.1177/1440783316635850

[bibr21-01430343221144407] Edwards S. (2015). Promoting family resilience in South Africa: A community psychological, multicultural counselling approach. *Inkanyiso*. Journal of Human and Social Science, 7(1), 38–43. https://hdl.handle.net/10520/EJC171656

[bibr22-01430343221144407] Evans S. Mikocka-walus A. Klas A. Olive L. Sciberras E. Karantzas G. Westrupp E. M. (2020). From “It has stopped our lives” to “Spending more time together – Has strengthened bonds”: The varied experiences of Australian families during COVID-19. Frontiers in Psychology, 11, 1–13. 10.3389/fpsyg.2020.588667 33192922 PMC7606874

[bibr23-01430343221144407] Fouché A. Fouché D. F. Theron L. C. (2020). Child protection and resilience in the face of COVID-19 in South Africa: A rapid review of C-19 legislation. Child Abuse & Neglect, 110, 104710. 10.1016/J.CHIABU.2020.104710 32938531 PMC7473143

[bibr24-01430343221144407] Gilligan M. Suitor J. J. Rurka M. Silverstein M. (2020). Multigenerational social support in the face of the COVID-19 pandemic. Journal of Family Theory and Review, 12(4), 431–447. 10.1111/jftr.12397 34367339 PMC8340915

[bibr25-01430343221144407] Gittings L. Toska E. Medley S. Cluver L. Logie C. H. Ralayo N. Chen J. Mbithi-Dikgole J. (2021). ‘Now my life is stuck!’: Experiences of adolescents and young people during COVID-19 lockdown in South Africa. Global Public Health, 16(6), 947–963. 10.1080/17441692.2021.1899262 33750269 PMC10105067

[bibr26-01430343221144407] Govender K. Cowden R. G. Nyamaruze P. Armstrong R. M. Hatane L. (2020). Beyond the Disease: Contextualized implications of the COVID-19 pandemic for children and young people living in Eastern and Southern Africa. Frontiers in Public Health, 8, 504. 10.3389/fpubh.2020.00504 33194933 PMC7604346

[bibr27-01430343221144407] Harrist A. W. Henry C. S. Liu C. Morris A. S. (2019). Family resilience: The power of rituals and routines in family adaptive systems. In Fiese B. H. Celano M. Deater-Deckard K. Jouriles E. N. Whisman M. A. (Eds.), APA Handbook of contemporary family psychology: Foundations, methods, and contemporary issues across the lifespan (pp. 223–239). American Psychological Association. 10.1037/0000099-013

[bibr28-01430343221144407] He W. Jia S. (2021). Exploring multigenerational co-residence in America[Paper presentation]. 20th International Business and Economy Conference, New York, January 4–8, 2021. https://www.ibec-info.org/2021/wp-content/uploads/2021/01/IBEC-2021-Conference-Proceedings-final-on-210118.pdf#page=119

[bibr29-01430343221144407] Iztayeva A. S. Sciences U. (2021). Custodial single fathers before and during the COVID-19 crisis: Work, care, and well-being. Social Sciences, 10, 94. 10.3390/socsci10030094

[bibr30-01430343221144407] Jimerson S. R. Stewart K. Skokut M. Cardenas S. Malone H. (2009). How many school psychologists are there in each country in the world?: International estimates of school psychologists and school psychologist-to-student ratios. School Psychology International, 30(6), 555. https://doi.org//10.1177/0143034309107077

[bibr31-01430343221144407] Lee S. J. Ward K. P. Lee J. Y. Rodriguez C. M. (2021). Parental social isolation and child maltreatment risk during the COVID-19 pandemic. Journal of Family Violence, 37, 813–824. 10.1007/s10896-020-00244-3 33462526 PMC7807402

[bibr32-01430343221144407] Loades M. E. Chatburn E. Higson-Sweeney N. Reynolds S. Shafran R. Brigden A. Linney C. McManus M. N. Borwick C. Crawley E. (2020). Rapid systematic review: The impact of social isolation and loneliness on the mental health of children and adolescents in the context of COVID-19. Journal of the American Academy of Child and Adolescent Psychiatry, 59(11), 1218–1239.e3. 10.1016/j.jaac.2020.05.009 32504808 PMC7267797

[bibr33-01430343221144407] Maree J. G. (2021). Managing the COVID-19 pandemic in South African Schools: Turning challenge into opportunity. South African Journal of Psychology, 52, 249–261. 10.1177/00812463211058398

[bibr34-01430343221144407] Mashaphu S. Talatala M. Seape S. Eriksson L. Chiliza B. (2021). Mental health, culture and resilience-Approaching the COVID-19 pandemic from a South African perspective. Frontiers in Psychiatry, 12, 955. 10.3389/FPSYT.2021.611108 PMC8292711 34305663

[bibr35-01430343221144407] Masten A. S. Motti-Stefanidi F. (2020). Multisystem resilience for children and youth in disaster. Adversity and Resilience Science, 1, 95–106. 10.1007/s42844-020-00010-w 32838305 PMC7314620

[bibr36-01430343221144407] Masten A. (2018). Resilience theory and research on children and families: Past, present, and promise. Journal of Family Theory and Review, 10(1), 12–31. 10.1111/jftr.12255

[bibr37-01430343221144407] Naidu T. (2020). The COVID-19 pandemic in South Africa. Psychological Trauma: Theory, Research. Practice, and Policy, 12(5), 559–561. 10.1037/TRA0000812 32525389

[bibr38-01430343221144407] October K. R. Petersen L. R. Adebiyi B. Rich E. Roman N. V. (2022). COVID-19 daily realities for families: A South African sample. International Journal of Environmental Research and Public Health, 19, 221. 10.3390/ijerph19010221 PMC8750582 35010480

[bibr39-01430343221144407] Panda P. K. Gupta J. Chowdhury S. R. Kumar R. Meena A. K. Madaan P. Sharawat I. K. Gulati S. (2021). A systematic review and meta-analysis is the psychological and behavioral impact of lockdown and quarantine measures for COVID-19 pandemic on children, adolescents and caregivers. Journal of Tropical Pediatrics, 67, 1–13. 10.1093/tropej/fmaa122 PMC7798512 33367907

[bibr40-01430343221144407] Parker C. Scott S. Geddes A. (2021). Snowball sampling other entries SAGE Research Methods Foundations. SAGE Research Methods Foundations. http://methods.sagepub.com/foundations/snowball-sampling

[bibr41-01430343221144407] Pernegger L. Godehart S. (2007). Townships in the South African geographic landscape – Physical and social legacies and challenges. http://www.treasury.gov.za/divisions/bo/ndp/ttri/ttri%20Oct%202007/Day%201%20-%2029%20Oct%202007/1a%20Keynote%20Address%20Li%20Pernegger%20Paper.pdf

[bibr42-01430343221144407] Pocock B. Williams P. Skinner N. (2012). Conceptualising work, family, and community: A socio-ecological systems model, taking account of power, time, space and life stage. British Journal of Industrial Relations, 50(3), 391–411. 10.1111/j.1467-8543.2011.00852.x

[bibr43-01430343221144407] Posel D. Casale D. (2021). Moving during times of crisis: Migration, living arrangements and COVID-19 in South Africa. Scientific African, 13, e00926. 10.1016/j.sciaf.2021.e00926 PMC10190999 37214590

[bibr44-01430343221144407] Posel D. Oyenubi A. Kollamparambil U. (2021). Job loss and mental health during the COVID-19 lockdown: Evidence from South Africa. PLoS ONE, 16, e0249352. 10.1371/journal.pone.0249352 33784339 PMC8009396

[bibr45-01430343221144407] Posel D. Casale D. (2020). Who moves during times of crisis? Mobility, living arrangements and COVID-19 in South Africa. https://cramsurvey.org/wp-content/uploads/2020/07/Posel-Who-moves-during-times-of-crisis_-Mobility-living-arrangements-and-COVID-19-in-South-Africa.-1.pdf 10.1016/j.sciaf.2021.e00926 PMC10190999 37214590

[bibr46-01430343221144407] Prime H. Wade M. Browne D. T. (2020). Risk and resilience in family well-being during the COVID-19 pandemic. American Psychologist, 75(5), 631–643. 10.1037/amp0000660 32437181

[bibr47-01430343221144407] Rider E. A. Ansari E. Varrin P. H. Sparrow J. (2021). Mental health and well-being of children and adolescents during the COVID-19 pandemic. British Medical Journal, 374, 1730. 10.1136/bmj.n1730 34429302

[bibr48-01430343221144407] Russell B. S. Hutchison M. Tambling R. Tomkunas A. J. Horton A. L. (2020). Initial challenges of caregiving during COVID-19: Caregiver burden, mental health, and the parent-child relationship. Child Psychiatry and Human Development, 51(5), 671–682. 10.1007/s10578-020-01037-x 32749568 PMC7398861

[bibr49-01430343221144407] Samji H. Wu J. Ladak A. Vossen C. Stewart E. Dove N. Long D. Snell G. (2022). Review: Mental health impacts of the COVID-19 pandemic on children and youth-a systematic review. Child and Adolescent Mental Health, 27(2), 173–189. 10.1111/camh.12501 34455683 PMC8653204

[bibr50-01430343221144407] Semo B. Frissa S. M. (2020). The mental health impact of the COVID-19 pandemic: Implications for Sub-Saharan Africa. Psychology Research and Behavior Management, 13, 713–720. 10.2147/PRBM.S264286 32982500 PMC7508558

[bibr51-01430343221144407] Shorey S. Ng E. D. (2022). A social-ecological model of grandparenting experiences: A systematic review. The Gerontologist, 62(3), e193–e205. 10.1093/geront/gnaa172 33146711

[bibr52-01430343221144407] Spinelli M. Lionetti F. Setti A. Fasolo M. (2021). Parenting stress during the COVID-19 outbreak: Socioeconomic and environmental risk factors and implications for children emotion regulation. Family Process, 60(2), 639–653. 10.1111/famp.12601 32985703

[bibr53-01430343221144407] Statistics South Africa (StatsSA). (2020). General housing survey. https://www.statssa.gov.za

[bibr54-01430343221144407] Stokols D. (1992). Establishing and maintaining healthy environments: Toward a social ecology of health promotion. American Psychologist, 47(1), 6–22. 10.1037/0003-066X.47.1.6 1539925

[bibr55-01430343221144407] Swart E. Eloff I. (2018). Educational psychology as science and profession in South Africa. In Eloff I. Swart E. (Eds.), Understanding educational psychology (pp. 2–9). Juta.

[bibr56-01430343221144407] Swart E. Pettipher R. (2016). A framework for understanding inclusion. In Landberg E. Kruger D. Swart E. (Eds.), Addressing barriers to learning (pp. 3–27). Van Schaik.

[bibr57-01430343221144407] The Lancet Public Health. (2020). Will the COVID-19 pandemic threaten the SDGs? The Lancet Public Health, 5(9), e460. https://www.ncbi.nlm.nih.gov/pmc/articles/PMC7462553/pdf/main.pdf 32888438 10.1016/S2468-2667(20)30189-4 PMC7462553

[bibr58-01430343221144407] Thibodeau-Nielsen R. B. Palermo F. White R. E. Wilson A. Dier S. (2021). Child adjustment during COVID-19: The role of economic hardship, caregiver stress, and pandemic play. Frontiers in Psychology, 12, 716651. 10.3389/fpsyg.2021.716651 34484078 PMC8416273

[bibr59-01430343221144407] Ting R. S. K. Aw Yong Y. Y. Tan M. M. Yap C. K. (2021). Cultural responses to COVID-19 pandemic: Religions, illness perception, and perceived stress. Frontiers in Psychology, 12(December 2019), 1–19. 10.3389/fpsyg.2021.634863 PMC8375556 34421700

[bibr60-01430343221144407] Uccella S. de Grandis E. de Carli F. D’Apruzzo M. Siri L. Preiti D. di Profio S. Rebora S. Cimellaro P. Biolcati Rinaldi A. Venturino C. Petralia P. Ramenghi L. A. Nobili L. (2021). Impact of the COVID-19 outbreak on the behavior of families in Italy: A focus on children and adolescents. Frontiers in Public Health, 9(February), 1–11. 10.3389/fpubh.2021.608358 PMC7893111 33614580

[bibr61-01430343221144407] Usher K. Bradbury Jones C. Bhullar N. Durkin D. J. Gyamfi N. Fatema S. R. Jackson D. (2021). COVID-19 and family violence: Is this a perfect storm? International Journal of Mental Health Nursing, 30(4), 1022–1032. 10.1111/inm.12876 34008291 PMC8242793

[bibr62-01430343221144407] Walsh F. (2016). Family resilience: A developmental systems framework. European Journal of Developmental Psychology. 10.1080/17405629.2016.1154035

[bibr63-01430343221144407] Walsh F. (2020). Loss and resilience in the time of COVID-19: Meaning making, hope and transcendence. Family Process, 59(3), 898–911. 10.1111/famp.12588 32678915 PMC7405214

[bibr64-01430343221144407] Wild L. G. (2018). Grandparental involvement and South African adolescents’ emotional and behavioural health: A summary of research findings. Contemporary Social Science, 13(2), 232–245. https://doi.org/1080/21582041.2017.1422536

[bibr65-01430343221144407] Woodley X. Lockard M. (2016). Womanism and snowball sampling: Engaging marginalized populations in holistic research. The Qualitative Report, 21(2), 321–329. 10.46743/2160-3715/2016.2198

[bibr66-01430343221144407] Zeng Z. Xie Y. (2014). The effects of grandparents on children’s schooling: Evidence from rural China. Demography, 51(2), 599–617. 10.1007/s13524-013-0275-4 24578167 PMC4026185

